# Metastable Materials Accessed under Moderate Pressure Conditions (P ≤ 3.5 GPa) in a Piston-Cylinder Press

**DOI:** 10.3390/ma14081946

**Published:** 2021-04-13

**Authors:** Javier Gainza, Federico Serrano-Sánchez, João Elias F. S. Rodrigues, Norbert Marcel Nemes, José Luis Martínez, José Antonio Alonso

**Affiliations:** 1 Instituto de Ciencia de Materiales de Madrid (ICMM), Consejo Superior de Investigaciones Científicas (CSIC), Sor Juana Inés de la Cruz 3, E-28049 Madrid, Spain; jgainza@ucm.es (J.G.); fserrano@icmm.csic.es (F.S.-S.); rodrigues.joaoelias@gmail.com (J.E.F.S.R.); martinez@icmm.csic.es (J.L.M.); 2 Departamento de Física de Materiales, Universidad Complutense de Madrid, E-28040 Madrid, Spain; nmnemes@fis.ucm.es

**Keywords:** high-pressure synthesis, RNiO_3_ perovskites, metal-insulator transitions, CaCu_3_Mn_4_O_12_, colossal magnetoresistance, SeNiO_3_, SeCoO_3_, NaMgH_3_, Tl_2_Mn_2_O_7_, CoSb_3_

## Abstract

In this review, we describe different families of metastable materials, some of them with relevant technological applications, which can be stabilized at moderate pressures 2–3.5 GPa in a piston-cylinder press. The synthesis of some of these systems had been previously reported under higher hydrostatic pressures (6–10 GPa), but can be accessed under milder conditions in combination with reactive precursors prepared by soft-chemistry techniques. These systems include perovskites with transition metals in unusual oxidation states (e.g., RNiO_3_ with Ni^3+^, R = rare earths); double perovskites such as RCu_3_Mn_4_O_12_ with Jahn–Teller Cu^2+^ ions at A sites, pyrochlores derived from Tl_2_Mn_2_O_7_ with colossal magnetoresistance, pnictide skutterudites M_x_Co_4_Sb_12_ (M = La, Yb, Ce, Sr, K) with thermoelectric properties, or metal hydrides Mg_2_MH_x_ (M = Fe, Co, Ni) and AMgH_3_ (A: alkali metals) with applications in hydrogen storage. The availability of substantial amounts of sample (0.5–1.5 g) allows a complete characterization of the properties of interest, including magnetic, transport, thermoelectric properties and so on, and the structural characterization by neutron or synchrotron X-ray diffraction techniques.

## 1. Introduction

Many interesting materials (oxides, chalcogenides, pnictides, hydrides) with exceptional electronic properties are metastable under ambient conditions and require special synthesis conditions such as high pressure, or the use of strongly oxidizing or reducing conditions or, in general, the utilization of moderate treatment temperatures. Some of these metastable materials, given the difficulty of their preparation, had been little studied so far, in spite of their interesting properties. This is the case, for example, of new oxides of transition metals in unusual oxidation states (e.g., V(IV), Cr(IV), Mo(V), Fe(IV), Ni(III), Cu(III)) or intermediate valence states (such as Cu(II)-Cu(III), Mn(III)-Mn(IV), Fe(II)-Fe(III)), some of which present strong electronic correlations that are responsible for interesting properties such as the superconductivity, the metallic behaviour and the metal-to-insulator transitions or the colossal magnetoresistance phenomenon. As a brief overview, the high hydrostatic pressure favours the formation of the short and strongly covalent chemical bonds characterizing the high oxidation states; on the other hand, the high pressure applied by a reactive gas such as oxygen provides the highly oxidizing conditions necessary for the stabilization of some of the mentioned valence states. Additionally, pressure prevents the decomposition of unstable reactants at the synthesis temperature (e.g., Tl_2_O_3_, CrO_2_); it helps to increase the coordination numbers and it favours the denser phases, in perovskite-like materials [1,2,3,4], skutterudites, pyrochlores and so on. Finally, high pressure enhances the reaction kinetics substantially. Thus, high pressure is a good choice to prepare new compounds with a low stability or a metastable character.

In the past 20 years, our group at the Instituto de Ciencia de Materiales de Madrid (CSIC) has been dealing with the stabilization of several families of metastable materials, which have been accessed with a simple piston-cylinder hydrostatic press, under moderate pressure conditions in the 2.0–3.5 GPa range. The synthesis of some of these systems was previously described to proceed at superior pressures (6–10 GPa); in our case the combination of wet chemistry procedures to obtain reactive precursors, or the choice of the reactants, was key to succeed under moderate pressure conditions. In the following we will describe some results corresponding to the families of RNiO_3_ perovskites (R = rare-earths), Tl_2_Mn_2_O_7_ pyrochlores and derivatives, CaCu_3_Mn_4_O_12_ double perovskites and derivatives, M_x_Co_4_Sb_12_ skutterudites (M = alkali, alkali earth or rare earth elements), (Se,Te)MO_3_ (M = Ni, Mn, Co) perovskites and AMgH_3_ hydrides (A = alkali metals).

## 2. Materials and Methods

**Figure 1:**
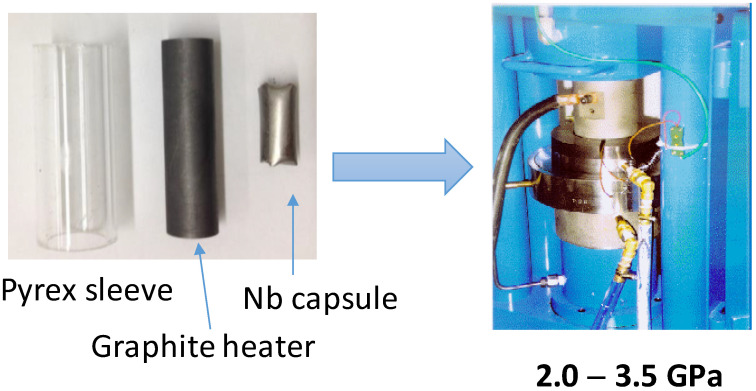
(**Left**): Graphite heater, Pyrex sleeve and Nb capsule (made of an Nb foil). (**Right**): view of the piston-cylinder press, including the power cable, thermocouple wiring and water cooling systems.

In all cases, the high-pressure reactions were carried out in a piston-cylinder Rockland Research press (Rockland Research Corporation, West Nyack, NY, USA), attaining maximum pressures of 3.5 GPa (piston of ½″) or 2 GPa (piston 3/4″). The samples were introduced in gold, platinum or niobium capsules, depending on the chemical nature of the reactants. These capsules were set in graphite cylinders acting as heaters, with Pyrex sleeves acting as pressure medium (Figure 1). The final pressure was applied in cold; the temperature was increased at 20 °C/min. Pressurization and depressurization rates were 2 GPa/h. After the heating period (20–60 min), the sample was rapidly cooled (100 °C/s) and then the pressure was slowly released. Therefore, the high-pressure products were quenched to a metastable state, where they were kinetically stable for long times. The reactants and experimental details for each system were as follows:

***RNiO_3_ perovskites.*** Stoichiometric mixtures of analytical grade Ni(OH)_2_ (99%, Aldrich Chemical Company Inc., St. Louis, MO, USA) and R_2_O_3_ (R = rare earths) (99.9%, Alfa Aesar, Karlsruhe, Germany) were ground in an agate mortar with 30% KClO_4_ (>99.5%, Fluka Chemika, Buchs, Switzerland), incorporated to provide an in situ high-oxygen pressure to promote the oxidation of nickel to Ni^3+^. The precursor mixture was introduced into a gold capsule of 5 mm diameter. A hydrostatic pressure of 2 or 3.5 GPa was applied; the sample was then heated at 900 °C for 20 min, followed by quenching. The KCl resulting from the decomposition of KClO_4_ and traces of R_2_O_3_ and NiO was subsequently eliminated by washing the resulting powder in a diluted HNO_3_ solution (65%, J.T. Baker, Schwerte, Germany) at 60 °C. The sample was then dried at 150 °C for 1 h in air.

***RCu_3_Mn_4_O_12_ double perovskite derivatives*.** Stoichiometric amounts of analytical grade, R_2_O_3_ (R = rare earths), Cu(NO_3_)_2_⋅3H_2_O and MnCO_3_ (99.9+%, Aldrich, Steinheim, Germany) were dissolved in citric acid. For R = Ce, Pr, Tb, Th, Ce(NO_3_)_3_, Pr_6_O_13_ or Tb_4_O_7_ or Th(NO_3_)_4_·5H_2_O were used, respectively, instead of R_2_O_3_. For Fe-substituted samples, FeC_2_O_4_ was used as reactant. The citrate solution turned into a resin by slow evaporation and drying at 120 °C. Afterwards, all the organic materials and nitrates were eliminated by heating at 600 °C for 12 h. This precursor was mixed with KClO_4_ (30% in weight) and thoroughly ground, sealed in a gold capsule (8 mm diameter, 10 mm length) and inserted in a cylindrical graphite heater. Hydrostatic pressure of 2 GPa and 1000 °C temperature were applied for 60 min to carry out the reaction. The in situ decomposition of KClO_4_ provides the high O_2_ pressure required to stabilize Mn^4+^ cations. Subsequently, the product was ground and washed in a dilute HNO_3_ aqueous solution, in order to eliminate small amounts of unreacted CuO and dissolve KCl remaining from the decomposition of KClO_4_; then the powder was dried in air at 150 °C for 1 h.

***(Se,Te)MO_3_ (M = Ni, Co, Mn) perovskites.*** A thoroughly ground stoichiometric mixture of TeO_2_/H_2_SeO_3_ and Ni(OH)_2_/CoO/MnO (99%, Alfa Aesar, Kandel, Germany) was sealed in a platinum capsule (6 mm dia.), and loaded in a cylindrical graphite heater. The reaction occurred at a pressure of 3.5 GPa at 850 °C for 1 h. After quenching to room temperature the pressure was released.

***Metal hydrides, Mg_2_MH_x_ (M = Fe, Co, Ni) and AMgH_3_ (A: Li, Na, K).*** Polycrystalline samples of these nominal compositions were synthesized from stoichiometric mixtures of LiH, NaH, KH, MgH_2_ and metal Fe, Co or Ni (Alfa Aesar, 99.9%, Kandel, Germany). The precursors were handled in inert atmosphere in an N_2-_filled glove box, since the reactants are highly sensitive to air and moisture. The reactants were mixed and ground in a mortar and then sealed in a gold capsule (8 mm diameter, 10 mm length), and loaded in a cylindrical graphite heater. The reactions occurred at moderate conditions of 2 GPa and 775 °C for short reaction times, less than 45 min, followed by quenching to room temperature under pressure and finally releasing the pressure slowly. The gold capsule with the reaction products was opened inside the glove box, yielding dense pellets, which were ground to perform the structural characterization.

***Tl_2_Mn_2_O_7_ pyrochlore derivatives*.** Tl_2_Mn_2_O_7_, Tl_2−x_Bi_x_Mn_2_O_7_ (x = 0.1, 0.2), Tl_2−x_Cd_x_Mn_2_O_7_ (x = 0.1, 0.2), and Tl_2_Mn_1.8_Sb_0.2_O_7_ pyrochlores were synthesized from Tl_2_O_3_, CdO/Bi_2_O_3_/Sb_2_O_3_ and MnO_2_ (Alfa Aesar, 99%, Germany) powders. The oxide mixtures were sealed in an 8 mm-diameter gold capsule, and loaded in a cylindrical graphite heater. The reaction occurred in a piston-cylinder press, at a pressure of 2 GPa at 1000 °C for 1 h.

***Skutterudites M_x_Co_4_Sb_12_ (M = alkali, alkali-earth, rare-earth elements).*** The samples with different compositions M_x_Co_4_Sb_12_ (M = K, Sr, Y, La, Ce, Yb, Mm (mischmetal)) have been prepared by a solid-state reaction under moderate temperature and pressure conditions. The reagents used were analytical grade KH, Sr, Y, La, Ce, Yb, Co and Sb in powder form. About 1.1 g of the starting elements was mixed according to the stoichiometric amount and sealed in a 5 mm dia. niobium capsule in an N_2_-filled glove box, which was placed inside the graphite cylinder used as a heater. Reactions were carried out at a pressure of 3.5 GPa at 800 °C for 1 h. Afterwards, the products were quenched, and the pressure was released.

In all cases, the nature of the resulting powder was assessed by laboratory X-ray diffraction (XRD) in a Bruker-AXS D8 diffractometer (Bruker-AXS, Karlsruhe, Germany) (40 kV, 30 mA), with Cu Kα radiation (λ = 1.5418 Å). NPD patterns collected at the high resolution D2B neutron diffractometer of ILL-Grenoble were used for structural refinement. Although only a relatively small amount of sample was obtained from the high-pressure experiments (about 0.5 g), good quality patterns could be collected with the high-flux mode and a counting time of 4 h. A wavelength of 1.594 Å was selected from a Ge monochromator. Synchrotron X-ray diffraction (SXRD) experiments were carried out in transmission mode on the BL04-MSPD beamline of the ALBA synchrotron (Barcelona, Spain) using the highest angular resolution mode as provided by the MAD setup [5]. The samples were sealed in 0.7 mm diameter quartz capillaries that were rotating during acquisition time to increase powder averaging. The beam energy was 28 keV (λ = 0.4427 Å) or 32 keV (λ = 0.38776 Å), selected to optimize absorption. The analysis of the diffraction data was performed by the Rietveld method, using the FullProf Suite [6]. The diffraction peak shape was defined with a pseudo-Voight function; a linear interpolation between points devoid of reflections was considered as background. The final refinement included the scale factor, zero-point shift, width and asymmetry parameters, unit-cell parameters, atomic positions, and isotropic or anisotropic displacement factors. No regions were excluded from the refinements.

## 3. Results and Discussion

### 3.1. RNiO_3_ Perovskites (R = Rare Earths)

**Figure 2:**
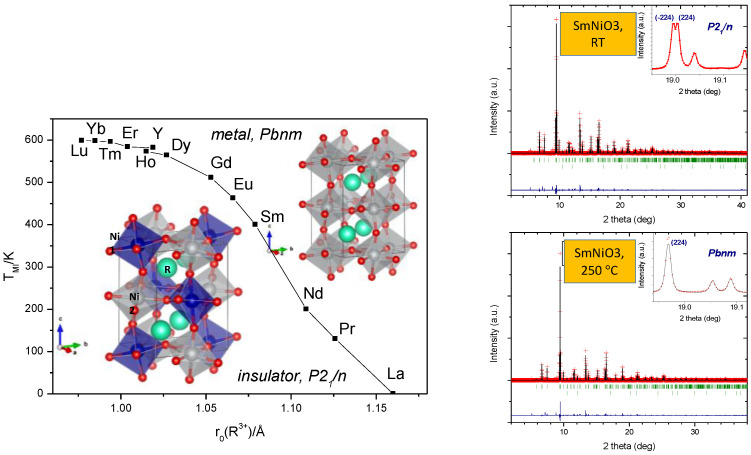
(**Left**): Phase diagram regarding the metal-insulator transitions experienced by RNiO_3_ perovskites as a function of temperature and the R^3+^ ionic size. The monoclinic structure corresponding to the insulating state (space group *P2_1_/n*) contains two types of crystallographically independent Ni atoms, whereas the orthorhombic structure (space group *Pbnm*) only contains one type of NiO_6_ octahedron. (**Right**): Characteristic splitting of the (2,2,4)/(-2,2,4) reflections observed by synchrotron X-ray diffraction (λ = 0.4427 Å), due to the monoclinic symmetry only in the insulator regime, below the MI transition in SmNiO_3_.

The detailed and systematic study of the metal-to-insulator (MI) transitions in the RNiO_3_ (R = rare earths) perovskites has been a long-standing topic, since these perovskites, containing Ni^3+^, are paradigmatic examples of charge-transfer materials that undergo abrupt metal-insulator transitions as a function of temperature and the size of the rare-earth cation. Torrance et al. [7] interpreted such transitions in terms of the reduction and closing of the charge-transfer gap between O^2−^ and Ni^3+^ ions. The presence of trivalent nickel makes it difficult to prepare these materials, particularly with smaller lanthanide radius. Thus, the transport properties (and MI transitions) had only been reported for R between La to Eu, prepared under high-oxygen pressures of 200 bar. There are examples of Ni perovskites synthesized at elevated pressures (~6 GPa) and temperatures (1000–1200 °C) in the literature [8,9,10], such as BiNiO_3_. However, the preparation of the phases for R = Y, Gd, Tb, Dy, Ho, Er, Tm, Yb, Lu, not synthesized again since the pioneering work by Démazeau in 1971 [11], was demonstrated to be possible using moderate pressures of 2 GPa. Sufficient amounts of samples were obtained to perform accurate neutron and synchrotron X-ray diffraction studies across the transitions. A charge disproportionation in YNiO_3_ associated with the MI transition was described for the first time [12,13]. In the monoclinic *P2_1_/n* crystal structure, Ni atoms occupy two independent crystallographic sites with slightly different charge, 3 + δ and 3 − δ, in such a way that there are alternating small and large octahedra along the 3 directions of the crystal (Figure 2).

This effect is extensive to other small rare-earth members, from R = Ho to R = Lu [14,15], for which the MI transition temperatures progressively increase as the R^3+^ size decreases (Figure 2). The access to these metastable materials made it possible to investigate a number of unexplored physical phenomena, which are mentioned hereafter: (i) High-pressure studies across the MI transitions by different techniques showed a metallization of the high-T phase for LuNiO_3_ [16]; (ii) Mössbauer results in ^57^Fe-doped Ni perovskites combined with neutron studies offered additional evidence of the charge disproportionation in RNiO_3_ (R = Sm, Eu, Gd, Dy) [17,18,19]; (iii) Transport measurements in sintered NdNiO_3_ yielded new features of the metallic state [20,21,22]; (iv) The investigation of absorbing DyNiO_3_ in double-walled sample holders allowed the establishment of charge disproportionation and the resolution of the magnetic structure of DyNiO_3_ [23]; (v) The observation, for the first time, of magnetic peaks by synchrotron soft X-ray resonant magnetic powder diffraction was possible in SmNiO_3_ samples [24]; and (vi) the study of the evolution of the charge transfer between Ni^3+δ^ and Ni^3−δ^ for the whole RNiO_3_ series was possible from X-ray absorption data [25].

More results concern (i) the study of magnetic and electronic properties of RNiO_3_ (R = Pr, Nd, Eu, Ho and Y) from resonant X-rays [26]; (ii) the magnetism and magnetic structures of the perovskite series NdNi_1−x_Mn_x_O_3_ from neutron powder diffraction [27]; (iii) Mössbauer results in ^57^Fe-doped Ni perovskites combined with neutron studies offering additional evidence of the charge disproportionation in RNiO_3_ (R = Tm and Yb) [28], and allowing us to complete the phase diagram for these materials (Figure 2); (iv) the stability of the Ni sites across the pressure-induced insulator-metal transition in YNiO_3_, previously prepared at 2 GPa [29]; and (v) the spin-canted magnetism and decoupling of the charge and spin-orbit coupling in NdNiO_3_ [30]. Recently, the extremely high angular resolution of the MSPD diffractometer at ALBA synchrotron X-ray source allowed us to observe the characteristic peak splitting in the (2,2,4) and (-2,2,4) twin reflections in SmNiO_3_ (Figure 2), thus confirming the monoclinic distortion and charge disproportionation in this perovskite with Sm^3+^ ion with a relatively large size [31].

### 3.2. CaCu_3_Mn_4_O_12_

Magnetoresistant (MR) materials experience an abrupt reduction in resistivity upon the application of an external magnetic field [32]. Its interest is induced, on one hand, by the wide panoply of technological applications in the field of magnetic sensors and read-head for magnetic disks. On the other hand, its study is also driven by more basic reasons, concerning the microscopic mechanisms that control these properties and, surprisingly, are related to other behaviours of the condensed matter such as the metallic state in oxides or the superconductivity.

In particular, we focus on a family of oxides that must be prepared under high-pressure conditions: the perovskites derived from CaCu_3_Mn_4_O_12_. This oxide is among the ferrimagnetic materials that have been “rediscovered” in light of their MR properties [33,34]. This complex perovskite with a Tc = 360 K, shows a considerable MR at low field: at room temperature MR is almost saturated for magnetic fields as low as 0.03 T. Therefore, its response upon an external field is much more abrupt than for other more conventional systems. In spite of these encouraging properties, this oxide has been very little studied due to its metastable character, which requires high-pressure preparation conditions. The synthesis of this family of materials had been described, up to now, to proceed at 5–6 GPa [33,35], yet we managed to synthesize CaCu_2.5_Mn_4.5_O_12_ at moderate pressures of 2 GPa, with comparable properties to those previously reported [36]. On the other hand, we have checked that the replacement of Ca for La or other rare-earths in RCu_3_Mn_4_O_12_ [37,38,39] has noticeable effects on the increment of T_C_, besides observing a significant increase of the low-field MR, up to 3% at RT in 1 T [40].

**Figure 3:**
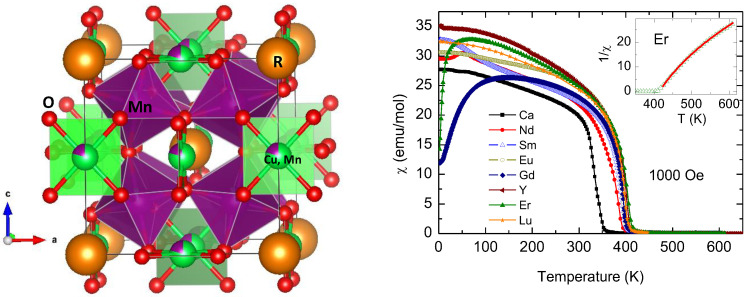
(**Left**): Crystal structure of (Ca,R)_3_Mn_4_O_12_, defined in the *Im-3* space group, with the MnO_6_ octahedra heavily tilted configuring a square planar oxygen coordination around Cu^2+^ (or Mn^3+^). (**Right**): Evolution of the Curie temperature for the long-range ferrimagnetic ordering of Cu^2+^ and Mn^4+^ spins, enhanced by the decrease of the R^3+^ ionic size. The inset shows the reciprocal susceptibility above T_C_ for the sample with Er, characteristic of ferrimagnetic systems.

These oxides can be considered as a 4-fold superstructure of perovskite (ABO_3_)_4_, with long-range order of R(Ca) and Cu at the A sites. The crystal structure exhibits the peculiarity of containing Cu^2+^ or Mn^3+^, which are Jahn–Teller ions [41], at the A positions of the perovskite, in square planar coordination, as shown in Figure 3. This is due to the strong tilting of MnO_6_ octahedra, defined in the *Im-3* cubic space group.

The peculiar magnetic structure of some members such as TbCu_3_Mn_4_O_12_ has also been studied by NPD [42]. There is an interesting correlation between some structural parameters and the magnetic properties, which was discovered from neutron or synchrotron X-ray diffraction studies of all the series. By replacing Ca^2+^ with R^3+^ cations in the parent CaCu_3_Mn_4_O_12_ oxide, there is a strong increase of the ferrimagnetic Curie temperature (T_C_), shown in Figure 3, due to electron injection. The ionic radii of R^3+^ cations from La to Lu along the rare-earth series decrease, creating an internal or chemical pressure: The accompanying compression of the MnO_6_ octahedral units with small rare earths brings about progressively shorter Mn-O distances and enhances the overlap between Mn and O orbitals, thus strengthening superexchange and increasing T_C_ by 50 K [43]. A transition from Pauli-paramagnetism to ferromagnetism was also observed in CaCu_3_(Ru_4−x_Mn_x_)O_12_ (0 ≤ x ≤ 3) perovskites [44], also prepared under moderate pressure conditions.

### 3.3. SeMO_3_ (M = Mn, Co, Ni)

In classical ABO_3_ oxides of the perovskite structure, at the A position usually an alkaline, alkaline-earth or rare-earth cation resides, with transition metals at the B sites. Much rarer are perovskite oxides containing *p-block* elements with an inert electron pair at the A positions, such as Tl^+^, Sn^2+^, Sb^3+^, Bi^3+^, Se^4+^ and Te^4+^. In order to stabilize perovskitelike oxides with these rather small cations, high-pressure conditions are typically required [1]. Two important examples are SeCuO_3_ [45] and BiMnO_3_ [46,47,48,49]. The large structural distortion bends the superexchange Cu-O-Cu or Mn-O-Mn angles and promotes the ferromagnetic interactions, according to the Goodenough–Kanamori rules [50]. We recently prepared and studied SeCoO_3_ [51], SeMO_3_ (M = Ni, Mn) [52], and TeNiO_3_ [53], which warrants the accessibility to these systems under moderate pressure conditions. There are several examples in the literature where this synthesis process has also been proven useful, such as the SeCo_1−x_Mn_x_O_3_ material prepared at ~4 GPa and ~1100 K for 1 h [54], and the SeMO_3_ (M = Mn, Ni) compounds synthesized at 3.5 GPa and 1123 K [55].

**Figure 4:**
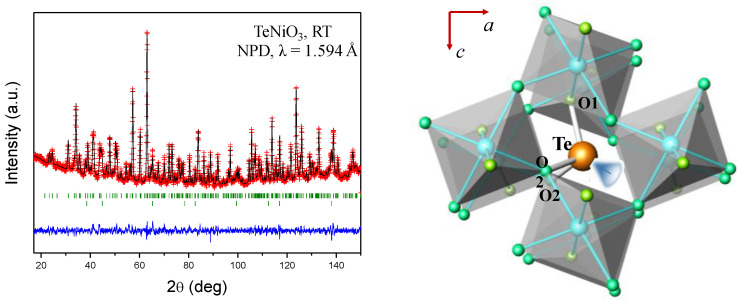
(**Left**): Neutron powder diffraction pattern of TeNiO_3_ perovskite, collected at D2B diffractometer (ILL-Grenoble) from a single-batch sample obtained at 3.5 GPa. A reactive mixture of Ni(OH)_2_ and TeO_2_, contained in a sealed platinum capsule under the reaction conditions (850 °C for 2 h), was essential to succeed. (**Right**): the extremely distorted environment for Te^4+^ ions leaves room for the electron lone pair of this p element, schematically represented in this structural view, highlighting the tilting of NiO_6_ octahedra.

In addition, the lone electron pair induces cationic shifts that favour ferroelectricity: together with their magnetism, these materials are magnetoelectric, useful for multiple-memory devices (storage of information by both electric and magnetic means) or memory elements written with electric field using its ferroelectricity but read magnetically as a ferromagnetic bit. In particular, in TeNiO_3_ [53] the extremely distorted crystal structure shapes a trigonal-pyramidal environment for the Te, where it is effectively coordinated to three oxygen atoms at Te–O distances of 1.92 Å (Figure 4). The 3-fold oxygen coordination is remarkably anomalous in perovskite-type structures, accounting for the required stabilization of these materials under high pressure conditions.

### 3.4. Metal Hydrides

The utilization of hydrogen as energy vector in the coming decades has boosted the research and improvement of hydrogen storage procedures. Although the use of compressed hydrogen is the immediate choice in electrical vehicles, safety considerations in case of a crash advise delving into the research of the ideal hydrogen storage material, still undiscovered. A classic example of metal hydride with hydrogen storage properties is LaNi_5_ and derivatives [56], able to form hydrides with composition LaNi_5_H_6_. New classes of materials composed of much lighter constituents (Li, Be, B, C, N, O, Na, Mg, Al, Si, P, S) have shown a much superior hydrogen storage capacity per weight than the conventional LaNi_5_ materials. Especially interesting are the Mg-based hydrides, given their high mass-storage capacity; as a drawback MgH_2_ is thermally stable, with decomposition temperatures above 450 °C, which prevents applications. The destabilization of MgH_2_ by doping with different metals has been an active research field in recent years.

**Figure 5:**
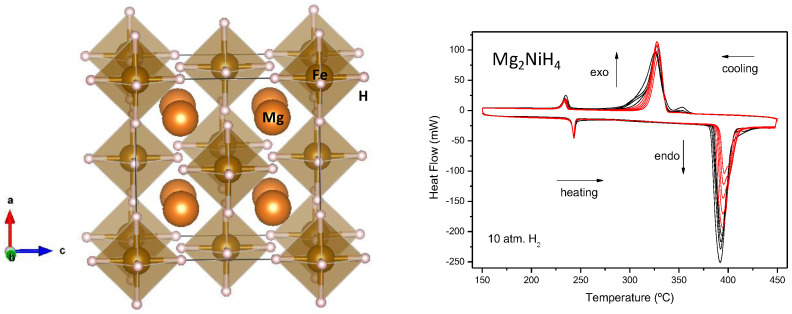
(**Left**): View of the crystal structure of Mg_2_FeH_6_, where Fe is octahedrally coordinated to 6 H atoms, while Mg is in 12-fold coordination, with Mg−H distances of 2.28 Å. (**Right**): Cyclic DSC curves of Mg_2_NiH_4_ in 10 bar of H_2_ at 10 °C min^−1^, showing a reduced temperature for H release with respect to MgH_2_ hydride, and a moderate reversibility. 20 cycles are shown; the endothermic peaks correspond to H_2_ release, and the exothermic ones to H_2_ uptake.

In this line, we have been successful in preparing several derivatives of the Mg_2_MH_x_ family (M = Fe, Co, Ni) [57,58] by direct reaction between the simple hydride MgH_2_ and the transition metals under high-pressure conditions, in gold capsules at 2 GPa. It is worth mentioning that Mg_2_FeH_6_ has one of the best H mass capacity ever described, almost 6%. Figure 5 shows the crystal structure of the novel Mg_2_FeH_6_ hydride, where Fe is octahedrally coordinated to hydrogen atoms. Figure 5 (right) also illustrates the cyclic hydrogen release-uptake of Mg_2_NiH_4_, prepared under moderate-pressure conditions.

This success in the preparative protocol has stimulated the synthesis of novel complex hydrides by direct reaction of simple hydrides under high-pressure conditions, which prevent the thermal decomposition of the reactants: we successfully prepared new hydride perovskites, of formula Na_1−x_Li_x_H_3_, by reaction under pressure of Na(Li)H and MgH_2_ [59], as well as Na_1−x_K_x_MgH_3_ [60]. In all these compounds, the location of H atoms, the study of the tilting of the BH_6_ octahedra, the presence of H vacancies and so on are also key knowledge to interpret the sorption/desorption kinetics.

**Figure 6:**
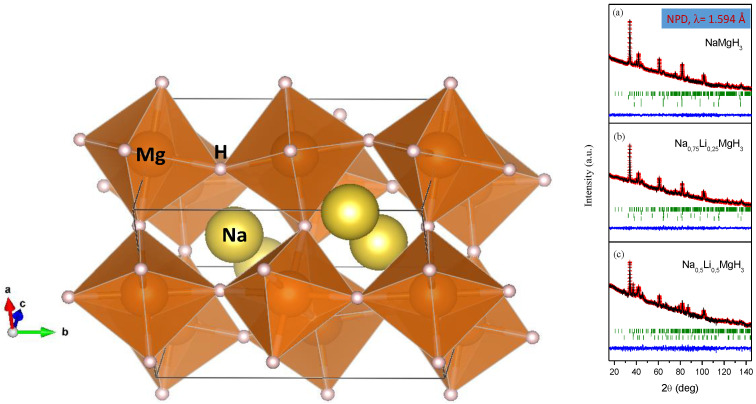
(**Left**): crystal structure of NaMgH_3_, exhibiting an orthorhombic superstructure of perovskite, space group *Pbnm*, with tilted MgH_6_ octahedra. (**Right**): neutron diffraction patterns for Na_1−x_Li_x_MgH_3_, displaying in the strong background the incoherent scattering of H. (**a**): x = 0; (**b**): x = 0.25; (**c**): x = 0.5.

Neutron diffraction techniques were powerful (and unique) tools to localize hydrogen atoms in these hydrides; Figure 6 shows the tilting of MgH_6_ octahedra in NaMgH_3_ perovskite, defined in the space group *Pbnm*, and exhibiting similar topology as the standard perovskite oxides [58]. This study demonstrated that it is not strictly necessary to study deuterated samples with neutrons, given the available flux in modern reactors or spallation sources.

### 3.5. Tl_2_Mn_2_O_7_

**Figure 7:**
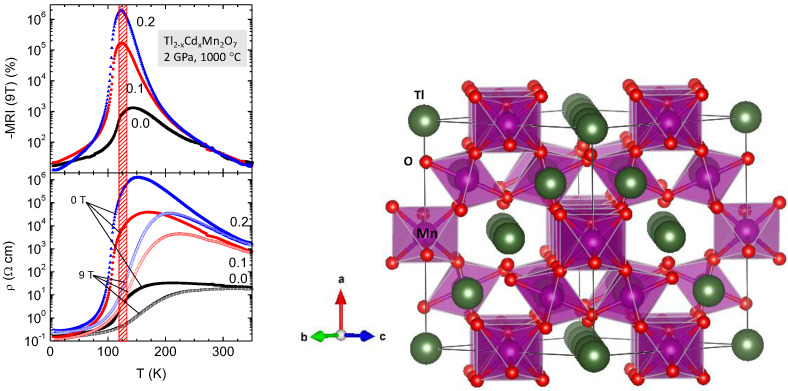
(**Left**): evolution of the magnetoresistance of the Tl_2−x_Cd_x_Mn_2_O_7_ family, showing maxima coinciding with the insulator-to-metal transitions (lower panel) in the 120–140 K temperature range. (**Right**): Structural view of the pyrochlore structure, defined in the cubic *Fd-3m* space group.

Tl_2_Mn_2_O_7_ pyrochlore, with tetravalent valence for Mn, does not seem to follow the conventional model of Double Exchange that is formally accepted for mixed-valence perovskites [61,62,63]; more research demonstrates that the magnetic superexchange may be mediated by the conduction electrons [64]. Tl_2_Mn_2_O_7_ is among the few metal oxides that simultaneously present ferromagnetic and metallic properties. This oxide is metastable at ambient conditions, and must be prepared under high-pressure conditions, at 2 GPa. Many efforts have been made to optimize the magnetoresistive properties of this singular material, in chemically doped derivatives. In addition, under moderate pressure conditions, diverse doped series were stabilized for the first time. The Tl sublattice of this pyrochlore has been doped with Bi, Pb and Cd, exhibiting very distinct physical properties. The Mn sublattice has been, alternatively, doped with Sb, Te and Ti [65,66,67,68]. In the Bi-doped phases, Tl_2−x_Bi_x_Mn_2_O_7_, with x = 0.05, 0.1, 0.2, 0.3 [65], the presence of competitive FM and AFM interactions gives rise to a “cluster-glass-type” behaviour, and promotes the magnetoresistance (MR) by several orders of magnitude above the undoped material. Moreover, the most spectacular results were obtained for the Cd-doped pyrochlore [66], with an MR up to 10^6^% at 2 K, and 20% at room temperature for magnetic field up to 0.5 T: this result has no precedents in the literature of magnetoresistive materials, and allowed proposing this material as a candidate for technological applications. Figure 7 illustrates the MR behaviour of Tl_2__−x_Cd_x_Mn_2_O_7_ pyrochlores, showing MR values up to 10^6^ for x = 0.2.

Also extremely interesting has been the study by neutron diffraction of the structural evolution under pressure of different doped pyrochlores, depending on the chemical nature of the different bonds, Tl-O and Mn-O, for the diverse studied families [69]. Figure 7 illustrates the crystal structure of the Tl_2_Mn_2_O_7_ pyrochlore, consisting of a framework of MnO_6_ octahedra sharing corners with Mn-O-Mn angles of about 133°, leaving large cages where Tl ions are located.

### 3.6. Skutterudites M_x_Co_4_Sb_12_ (M = Alkali, Alkali-Earth, Rare-Earth Elements)

Globally, about two-thirds of energy is dissipated as waste heat, providing an important niche for thermoelectric materials (TM). TMs can directly transform a temperature difference into electric energy [70,71,72]. However, a good TM must possess three antagonistic properties, hindering the design and development of novel materials: it requires a high Seebeck voltage (S, thermoelectric power), low electrical resistivity (ρ) and low thermal conductivity (κ). Most materials have a coupled thermal and electronic conductivity, since the same carriers are involved in both mechanisms to a large extent. TMs are characterized by a Figure of Merit (ZT) defined as (ZT = S^2^T/ρκ); the best ZT is around 1–2, depending on the T range. To date, the best TMs are strongly doped semiconductors such as PbTe or Bi_2_Te_3_.

**Figure 8:**
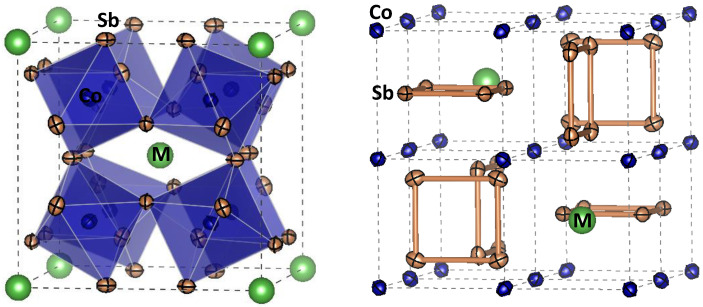
Two views of the skutterudite structure M_x_Co_4_Sb_12_ (M = filler element), defined in the cubic *Im-3* space group. The strongly tilted CoSb_6_ octahedra (**left** view) determine the presence of Sb_4_ rings (**right** view), of importance in the electronic properties. The M filler elements (alkali, alkali-earths, rare-earths) occupy the large 8a voids, where the rattling diminishes the thermal conductivity.

The possibility of preparing thermoelectric specimens under HP conditions was recently demonstrated [73,74,75,76], in particular in skutterudites. MX_3_ skutterudites derive their name from Skutterud, a small town in Norway, where a mineral based on CoAs_3_ was discovered in 1845, and their structure can host different transition metals (Fe, Co, Rh, Ir) at M position, and p-block semimetals (P, As, Sb) as X atoms [72]. A prominent feature is the existence of two relatively large voids that can be filled with additional atoms. Filled skutterudites contain lanthanide, or alkali-earth ions interstitially occupying these voids [77], with large thermal vibration parameters indicating that they can “rattle” or participate in a soft phonon mode of the crystal structure. This “ball in a cage” configuration of filled skutterudites directly determines the basic conditions for high ZT values. The effect of the atomic void fillers in oversized cages drastically reduces κ and thereby maximizes ZT [78,79]. The stabilization of such metastable compounds is possible, for example, for the parent, unfilled CoSb_3_ pnictide, which can be successfully stabilized at 3.5 GPa, with exceptionally low thermal conductivities that were ascribed to partial Sb deficiency, the Sb vacancies acting as phonon scatterers [80]. We also stabilized La_1−x_CoSb_3_, with different rattling La contents, optimizing the charge carrier concentration [81], as well as Ce, Yb [82] and even with Mischmetal [83] as rattling elements (cocktail of Ce, La and other rare earths) also at 3.5 GPa. A key finding to succeed in these HP syntheses was the use of capsules made of Nb sheets, instead of the conventional Au or Pt capsules: Nb is inert with respect to Sb, and the handmade cylinders are perfectly sealed under pressure, avoiding contact with air, which would oxidize this element or the rare-earth metal fillers. There are several additional works in the literature related to the high-pressure synthesis of skutterudites, but most of them are performed at very high pressures (~6–8 GPa) [84] or in several steps [85,86,87,88]. Figure 8 shows a view of the cubic M_x_Co_4_Sb_12_ skutterudite structure, defined in the cubic space group *Im-3*, containing strongly rotated CoSb_6_ octahedra (Co-Sb-Co ~126°) that, in fact, is very similar to the CaCu_3_Mn_4_O_12_ crystal structure (Figure 3), defined in the same space group, and also containing tilted MnO_6_ octahedra. In the present case, the strong rotation in the space conforms square Sb_4_ units (see Figure 8) that develop important role as traps for phonons, thus reducing the thermal conductivity.

In La-filled skutterudites, it has been additionally discovered that HP reactions favour fluctuations in the filling fraction, leading to glas-slike ultralow thermal conductivity in caged skutterudites [81]. This is in line with recent discoveries by ab initio calculations and synthesis of multiscale filling-fraction fluctuation in the RFe_4_Sb_12_ system [89]. HP brings about the separation into phases higher and lower La filler contents, leading to multiscale strain field fluctuations. The filling fraction fluctuation reduces the lattice thermal conductivity, via strain-field scattering of high-energy phonons. This strategy of favouring the uneven filling factor under HP conditions has been extended to other elements, such as K, Sr, Y and more [90].

## 4. Conclusions

Moderate-pressure conditions have been demonstrated to be a powerful tool for the stabilization of metastable materials with technological applications, sometimes in combination with a pertinent choice of the reactants or by elaborating suitable precursors by wet-chemistry procedures. Systems as different as transition-metal oxides in unusual oxidation states, metal pnictides or hydrides can be accessed in substantial amounts enabling a complete characterization. Besides favouring the short bonding distances of high oxidation states in densely packed structures such as perovskites, the reactions occur in laboratory-made sealed capsules avoiding the oxidation (e.g., Sb), or volatilization (e.g., SeO_2_), or decomposition (e.g., NaH, MgH_2_) of certain reactants, making it possible for the desired reactions in short times and controlled atmospheres (e.g., high O_2_ pressure). Those findings are to be applied in novel metastable systems as upcoming research directions: Some oxides with unusual oxidation states (e.g., Fe^4+^, in (Ca,Sr)FeO_3_ derivatives), and novel pnictides and chalcogenides such as SnSe derivatives and MM’_2_S_4_ (M, M’ = transition metals) thiospinels, accessible under moderate-pressure conditions, are to be exploited in the near future.

## Data Availability

No new data were created or analyzed in this study. Data sharing is not applicable to this article.
